# Analysis of risk factors for recurrence within 6 months after bronchial artery embolization for massive hemoptysis due to pulmonary tuberculosis

**DOI:** 10.1097/MD.0000000000041734

**Published:** 2025-03-07

**Authors:** Jiao Xu-Dong, Wang Qi-Fan, Shi Ya-Rong, Li Yun-Hua

**Affiliations:** a Department of Radiology, Wuxi Branch of Ruijin Hospital Shanghai Jiao Tong University School of Medicine (Wuxi Xinwu District Xinrui Hospital), Wuxi City, Jiangsu Province, China; b Department of Radiology, Jiangsu Province Changzhou 3rd Hospital, Jiangsu, Changzhou, China.

**Keywords:** bronchial artery embolization, massive hemoptysis, multivariate analysis, pulmonary tuberculosis, recurrence, risk factors

## Abstract

This study aims to investigate the causes and risk factors of recurrence within 6 months after transcatheter bronchial artery embolization for massive hemoptysis in pulmonary tuberculosis. A retrospective observational study was conducted on the clinical data of 237 patients with pulmonary tuberculosis and massive hemoptysis who relapsed within 6 months after bronchial artery embolization. Univariate analysis was first conducted to screen for risk factors with statistical significance, and then multivariate logistic regression analysis was performed to infer the risk factors that might lead to the recurrence of hemoptysis. Twenty-four relapsed within 6 months after the operation, and the recurrence rate was 10.1% (24/237). Logistic multivariate regression analysis revealed that 6 risk factors were included in the regression model. The strength of the effect was as follows: postoperative lesion progression (odds ratio [OR] = 4.429, 95% confidence interval [CI]: 2.833–7.283), number of feeding arteries (OR = 2.592, 95% CI: 1.457–4.741), combined bronchiectasis (OR = 2.324, 95% CI: 1.912–5.682), presence of cavitation (OR = 1.933, 95% CI: 1.406–2.451), systemic-to-pulmonary shunt (OR = 1.596, 95% CI: 1.044–2.409), and lesion lobe distribution (OR = 1.056, 95% CI: 1.006–1.128). Postoperative lesion progression, a large number of feeding arteries, combined bronchiectasis, presence of cavities, existence of a systemic-to-pulmonary shunt, and wide lesion distribution are independent risk factors for postoperative recurrence.

## 1. Introduction

Pulmonary tuberculosis (TB) complicated with acute and recurrent massive hemoptysis can easily cause asphyxia in patients, and severe cases can even lead to hemorrhagic shock. Bronchial artery embolization (BAE) has been widely accepted and adopted clinically because of its definite hemostatic effect. However, the recurrence of hemoptysis after BAE, especially in the short term (within 6 months), is a noticeable issue. Literature reports show that the recurrence rate of hemoptysis after bronchial artery embolization is 15% to 20%.^[1,2]^ We retrospectively investigated the clinical data of 237 patients with pulmonary TB and massive hemoptysis who underwent BAE treatment between October 2015 and May 2023. A retrospective observational study was conducted on 24 patients with recurrent hemoptysis within 6 months after to determine the main risk factors of recurrence after BAE to provide a theoretical basis for preventing short-term recurrence of massive hemoptysis after bronchial artery embolization in pulmonary TB.

## 2. Materials and methods

### 2.1. Clinical data

Among the 237 patients with pulmonary TB and massive hemoptysis, 149 were male and 88 were female; the age ranged from 19 to 74 years, with a median age of 54 years. Among the 24 patients in the recurrent case group, 15 were male and 9 were female, aged 23 to 67 years old, with an average age of 43 years. The basic lesion was TB in all cases, with more than 200 mL of hemoptysis at onset. All cases were ineffective, recurrent, or suddenly aggravated after conservative hemostasis treatment with medicine for 2 to 7 days in the Department of Internal Medicine and received selective BAE treatment. The characteristics of 237 patients are shown in Table 1.

**Table 1:** The characteristics of 237 patients.

Clinical data	Number & percentage
Gender distribution	Male (143, 60%), female (94, 40%)
Age	Range: 21–73 years, median: 54 years
Hemoptysis volume (distribution)	Range: 200–400 mL, median: 310 ± 20 mL
Tuberculosis status	Active (141, 59.5%), inactive (96, 40.5%)
Relevant comorbidities	Cavities (153, 64.5%), bronchiectasis (78, 32.9%), merge bacterial infections (65, 27.4%)
Target vessels	Multiple (67, 28.2%), single (170, 71.8%)
The types of embolic agents	Gelatin sponge granule (189, 79.7%), embolic coil (20, 8.5%), polyvinyl alcohol granule (28, 11.8%)
Primary disease after the BAE procedure	Worsened (36, 15.2%), improvement or stability (201, 84.8%)

BAE = bronchial artery embolization.

Twenty-four patients experienced short-term recurrence of hemoptysis within 6 months after BAE, including 15 males and 9 females aged 23 to 67 years with an average age of 43 years. Among them, 15 cases (63%) were active TB, and 9 cases (37%) were past TB sequelae.

### 2.2. Observation indicators

Based on previous clinical cases, experience summaries, and literature reports,^[2,3]^ to avoid missing some important factors, 11 factors were initially selected, including the age, gender, amount of hemoptysis, number of feeding arteries, arteriovenous shunt, distribution of lesion lung lobes, combined with bronchiectasis, postoperative lesion progression, accompanied by cavity, combined bacterial infection, and type of embolic agent of the patients.

### 2.3. Data processing and analysis

With the presence or absence of recurrent massive hemoptysis within 6 months after BAE for pulmonary TB massive hemoptysis as the dependent variable (Y: No = 0; Yes = 1), and the above 11 factors as independent variables. Univariate logistic regression analysis was conducted. Multivariate logistic analysis was performed on variables with *P* < .10. All independent variables were converted into categorical variables and introduced into the model as dummy variables. Statistical software (SPS 23.0) was used for processing. The forward stepwise regression method (Forward: Conditional) was used to screen the independent variables. The inclusion criterion was *P* = .05, and the removal criterion was *P* = .10. The relative risk was estimated by the odds ratio (OR) and 95% confidence interval (CI), and a logistic regression model was constructed.

## 3. Results

### 3.1. Angiographic manifestations and therapeutic efficacy observation

**Figure 1.**
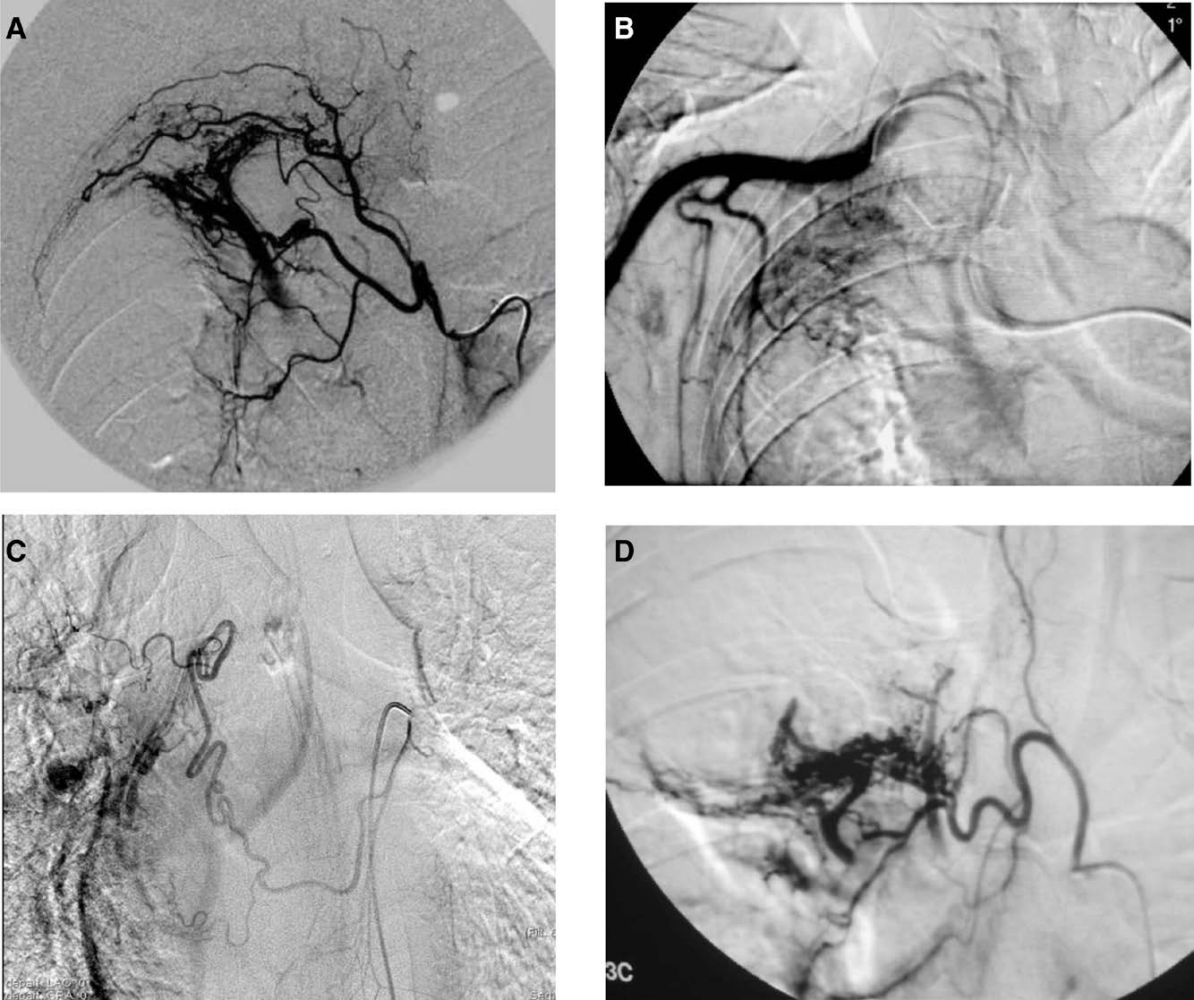
When there are multiple target vessels, the likelihood of incomplete embolization increases. Additionally, the greater the number of treated vessels, the higher the probability that one of them will recanalize. Besides, existence of a systemic-to-pulmonary shunt is one of risk factors. (A) The bronchial artery and the first intercostal artery share the same trunk, the bronchial artery is twisted and thickened, the contrast agent overflows, and a bronchial artery-pulmonary vein fistula is observed. (B) The lateral thoracic artery also participates in the blood supply of the lesion. (C) Angiography shows contrast agent extravasation. (D) Angiography shows distortion and thickening of bronchial arteries, reticular and lacunar changes of blood vessels in the lesion area, and bronchial artery-pulmonary vein fistula, with obvious visualization of the right superior pulmonary vein.

All bleeding vessels showed varying degrees of distortion, thickening, hyperplasia, and aneurysmal dilation (Fig. 1A), multiple target vessels (Fig. 1B), and some cases showed contrast agent extravasation (Fig. 1C), bronchial artery-pulmonary vein fistulas, and/or bronchial artery-pulmonary artery fistulas (Fig. 1D). All patients achieved immediate hemostasis; however, 24 had recurrent hemoptysis within 6 months. Among them, 11 patients had a bleeding amount of 50 mL/24 hours ≤ 100 mL, and 13 cases had a bleeding amount > 100 mL/24 hours. Among the 24 recurrent patients, 17 underwent secondary embolization, 4 underwent tertiary embolization, and no re-bleeding occurred during the follow-up for half a year; 3 patients underwent surgical lobectomy.

### 3.2. Unconditional logistic analysis

#### 3.2.1. Univariate analysis

The selected 11 variables were used for univariate analysis and logistic regression for variable screening (see Table 2). To avoid missing some important factors, the *P*-value was relaxed to .10 in the univariate analysis. The results showed that 8 factors, including the amount of hemoptysis, number of feeding arteries, systemic-to-pulmonary shunt, distribution of lesion lung lobes, combined with bronchiectasis, postoperative lesion progression, accompanied by cavity, and combined bacterial infection, were associated with recurrent massive hemoptysis (see Table 3).

**Table 2:** Possible risk factors and quantitative assignment for recurrence within 6 months after BAE for massive hemoptysis due to pulmonary TB.

Independent variables (observation indicators)	Assignment method	
Gender (X1)	Female (0)	Male (1)
Age (X2)	<20 years old (0)	20–29 years old (1)
	30–39 years old (2)	≥40 years old (3)
The amount of hemoptysis (X3)	<500 mL/d (0)	≥500 mL/d (1)
The number of feeding arteries (X4)	Single (0)	Multiple (1)
Arteriovenous shunt (X5)	Yes (0)	No (1)
Lesion lobe distribution (X6)	1–2 lobes (0)	3 lobes (1) 4 lobes and above (2)
Combined bronchiectasis (X7)	Yes (0)	No (1)
Associated with cavity lesion (X8)	Yes (0)	No (1)
Combined bacterial infection (X9)	Yes (0)	No (1)
Type of embolic agent (X10)	Gelatin sponge granule (0)	Embolic coil (1) polyvinyl alcohol granule (2)
Postoperative lesion progression (X11)	Yes (0)	No (1)

BAE = bronchial artery embolization, TB = tuberculosis.

**Table 3:** Univariate logistic regression analysis of recurrence within 6 months after BAE for massive hemoptysis due to pulmonary TB.

Independent variables	B	OR	95% CI	*P*-value
X1	1.528	1.485	1.074–2.591	.370
X2	0.967	1.737	1.440–2.132	.209
X3	0.597	1.857	1.366–3.674	.086
X4	0.680	1.605	1.017–1.855	.031
X5	0.934	2.349	1.631–3.575	.000
X6	0.572	1.559	1.083–2.029	.022
X7	0.597	1.560	1.266–1.857	.007
X8	0.540	2.604	1.382–3.256	.037
X9	0.073	1.156	1.016–1.928	.092
X10	0.081	1.126	1.051–1.415	.287
X11	1.763	3.234	2.687–5.682	.000

BAE = bronchial artery embolization, TB = tuberculosis.

#### 3.2.2. Multivariate analysis and predictive models

Variables with *P* < .10 from the univariate logistic analysis were entered into the equation for multivariate logistic analysis. Using the method of forcing independent variables into the regression model and adjusting, 6 risk factors were found to enter the regression model. In order of their strength of effect, postoperative lesion progression, the number of feeding arteries, bronchiectasis, accompanied by cavity, systemic-to-pulmonary shunt, and distribution of lesion lung lobes were observed (see Table 4). The obtained logistic regression prediction model is: *P* = e(-5.304 + 2.592*X4 + 1.596*X5 + 1.056*X63 + 2.324*X7 + 1.933*X8 + 4.429*X11)/(1 + e(-5.304 + 2.592*X4 + 1.596*X5 + 1.056*X63 + 2.324*X7 + 1.933*X8 + 4.429*X11)).

**Table 4:** Multivariate logistic regression analysis of recurrence within 6 months after BAE for massive hemoptysis due to pulmonary TB.

Independent variable	B	OR	95% CI	*P*-value
X4	1.093	2.592	1.457–4.741	.016
X5	0.076	1.596	1.044–2.409	.000
X6	0.072	1.056	1.006–1.128	.043
X7	2.236	2.324	1.912–5.682	.000
X8	0.569	1.933	1.406–2.451	.030
X11	1.745	4.429	2.833–7.283	.011
Constant	-5.304	0.627		.041

BAE = bronchial artery embolization, TB = tuberculosis.

According to this regression model, the possible risk factors for recurrent massive hemoptysis within 6 months after BAE, based on their strength, are as follows: postoperative lesion progression (OR = 4.429, 95% CI: 2.833–7.283), number of feeding arteries (OR = 2.592, 95% CI: 1.457–4.741), combined bronchiectasis (OR = 2.324, 95% CI: 1.912–5.682), presence of cavitation (OR = 1.933, 95% CI: 1.406–2.451), systemic-to-pulmonary shunt (OR = 1.596, 95% CI: 1.044–2.409), and lesion lobe distribution (OR = 1.056, 95% CI: 1.006–1.128).

## 4. Discussion

The main reasons for short-term hemoptysis recurrence after BAE include factors related to surgical techniques, as well as the progression and complications of the disease.

### 4.1. Technical factors for recurrence within 6 months after BAE for massive hemoptysis of pulmonary TB

#### 4.1.1. Multiple blood supplies of the lesion leading to incomplete embolization

When there are multiple target vessels, the likelihood of incomplete embolization increases. Additionally, the greater the number of treated vessels, the higher the probability that one of them will recanalize, thus increasing the overall risk of recurrence. The non-bronchial arteries involved in hemoptysis in the systemic arteries include intercostal arteries, internal thoracic arteries, thyrocervical trunks, inferior phrenic arteries, mediastinal branches of the thoracic aorta, etc.^[3]^ These non-bronchial arteries may be missed and not embolized during the embolization process or form collateral circulation after bronchial artery embolization and become a new source of bleeding, causing recurrent bleeding. During embolization treatment, in addition to bilateral bronchial arteriography, angiography of the intercostal arteries, subclavian arteries, etc. on the affected side should also be performed. Non-bronchial arteries involved in the blood supply should be sought and discovered as much as possible. The equation shows that, if missed, it is likely to lead to early recurrence of hemoptysis after embolization. It is generally believed that the intercostal arteries are involved in supplying blood to the parietal pleura. When the pleura is diseased, the intercostal arteries can penetrate the thickened and adherent pleura, proliferate abundantly in the lungs, and participate in the blood supply of intrapulmonary lesions. Therefore, for lesions close to the pleura and possibly involving the pleura, attention should be paid to the angiographic observation of multiple intercostal arteries. Thoracic aortic angiography is useful for monitoring therapeutic efficacy after embolization technique. In addition, because bronchial artery embolization can only control massive bleeding from bronchial arteries and cannot control bleeding from pulmonary arteries and destructive lesions of the lungs can erode adjacent systemic and pulmonary circulation, hemoptysis will continue after bronchial artery embolization.^[4]^

#### 4.1.2. Existence of systemic-to-pulmonary shunt

Some bronchial arteries anastomose with the pulmonary circulation at the level of the respiratory bronchi, especially when there is a large systemic-to-pulmonary shunt. To avoid emboli entering the pulmonary veins, larger embolic materials must be selected, which may result in incomplete embolization of bleeding vessels, especially smaller peripheral vessels. This undoubtedly poses the risk of continued hemoptysis after embolization.

#### 4.1.3. Selection of embolic agents and embolization methods

There are many materials available for embolization therapy of bronchial arteries, such as gelatin sponge granule, embolic coil, polyvinyl alcohol granule, and embospheres. Embolic coil is a permanent embolization agent suitable for large blood vessels or main arteries, but peripheral blood vessels such as Intercostal artery, internal thoracic artery, lateral thoracic artery may form collateral circulation; polyvinyl alcohol granules are suitable for peripheral vascular occlusion, but the granules may block proximal blood vessels and pose a higher risk of nontarget occlusion. Embospheres can accurately embolize small blood vessels, but the cost is high and may increase the risk of pulmonary infarction. Gelatin sponges granule are widely used clinically because of their low price. However, it can be absorbed within a certain period (7–14 days). To reduce the occurrence of such situations, we adopted the method of multiple embolizations of the same bleeding vessel and performing 2 rounds of high-pressure treatment on the gelatin sponge to make it harder.

The literature shows that the short-term recurrence rate of hemoptysis with different embolic agents^[5,6]^ is as follows: gelatin sponge granule > embolic coil ≈ polyvinyl alcohol granule. However, in the univariate analysis of this group, the embolic agent variable was excluded, and the reason for the analysis may be that gelatin sponge granule was mainly used in this group, and the sample size of other embolic agents was too small.

### 4.2. Non-technical factors for recurrence of massive hemoptysis due to pulmonary TB within 6 months after BAE

After multi-factor logistic regression analysis, we found that the progression of the lesion after BAE had the greatest impact on the recurrence of hemoptysis. Technical factors such as missed embolization and involvement of extrapulmonary systemic circulation in providing blood supply to intrapulmonary lesions result in incomplete vascular treatment in patients with postoperative hemoptysis recurrence after embolization for TB, mainly because of the persistence of chronic infectious inflammation within the TB lesion, various secondary chronic pulmonary infections, and recurrence of TB.^[7,8]^ After emergency interventional embolization, active internal medical treatment should be combined, including hemostasis and antituberculosis treatment. Progress of the primary lesion or persistence of infection on the basis of the inherent lesion can cause vascular regrowth, dilation, rupture, and bleeding. Repeated inflammatory stimulation may dissolve and recanalize emboli in embolized vessels, resulting in the recurrence of hemoptysis.

#### 4.2.1. Combination of bronchiectasis, cavities and extensive lesion distribution

**Figure 2.**
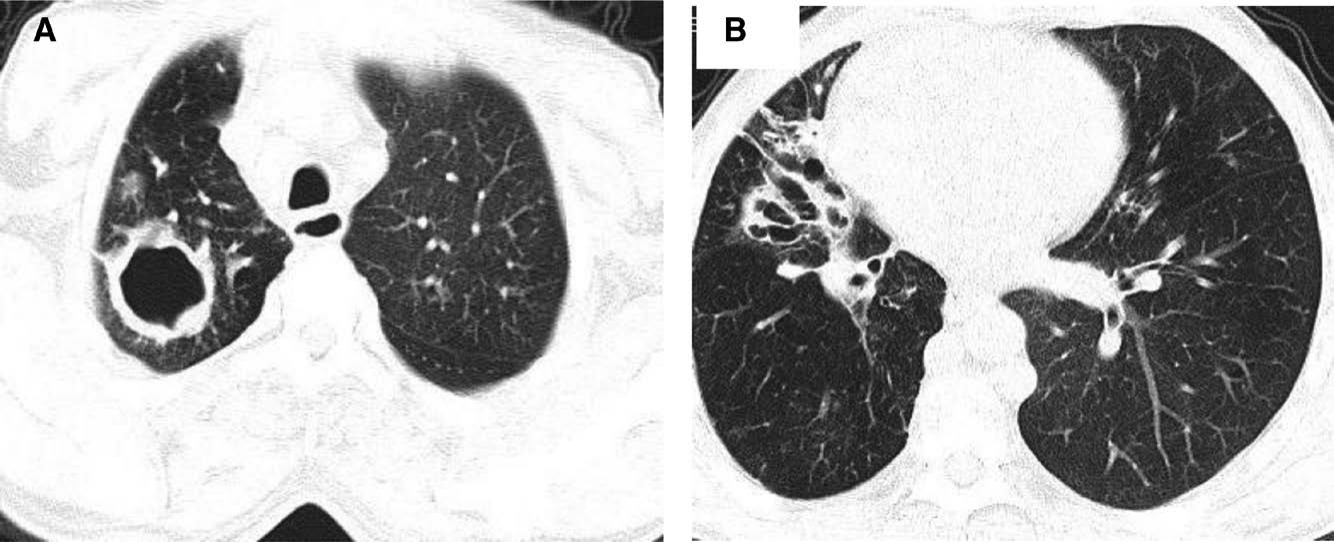
Progress of the primary lesion can cause vascular regrowth, dilation, rupture, and bleeding. Repeated inflammatory stimulation may dissolve and recanalize emboli in embolized vessels. Bronchiectasis is prone to repeated combined infectious inflammation, resulting in new vascular growth, dilation, rupture and bleeding. (A) There is a cavity lesion in the apical segment of the right upper lobe of the lung in tuberculosis. (B) For the same patient as (A), accompanied by cystic dilation of the bronchus in the right middle lobe at the same time.

In this group study, it was concluded that the more severe the lesion of pulmonary TB, the greater the chance of hemoptysis recurrence after BAE. The reasons for the high recurrence rate after BAE in patients with combined bronchiectasis, cavities (Fig. 2), or extensive lesion distribution with fibrosis may be that the degree of lung damage is large, the blood supply in areas with severe fibrosis and calcification is abundant, and the collateral circulation will be established quickly after the old vessels are embolized, becoming a new source of bleeding^[8,9]^; resistance to antituberculosis drugs and bronchiectasis is prone to repeated combined infectious inflammation, resulting in new vascular growth, dilation, rupture and bleeding, and recurrence of hemoptysis; post-embolization chest pain causing shortness of breath and coughing may also be the cause of vessel rupture again in the short term.^[9,10]^

In conclusion, the progression of the lesion after BAE, large number of feeding arteries, bronchiectasis, presence of cavities, existence of systemic-to-pulmonary shunt, and extensive lesion distribution are independent risk factors for recurrence. There are numerous causes of short-term recurrence after BAE, and the conditions are complex. The risk model proposed in this study is only a reference basis, and the sample size of recurrent patients in this group was insufficient. The observation and evaluation of risk factors or predictors in the above model need to be further tested and improved in clinical practice.

## Author contributions

**Data curation:** Jiao Xu-Dong.

**Investigation:** Jiao Xu-Dong, Wang Qi-Fan, Shi Ya-Rong, Li Yun-Hua.

**Methodology:** Jiao Xu-Dong, Wang Qi-Fan.

**Project administration:** Jiao Xu-Dong.

**Writing – original draft:** Jiao Xu-Dong, Wang Qi-Fan.

**Writing – review & editing:** Jiao Xu-Dong.
